# Genome Sequence of a Rabies Virus Isolated from a Dog in Chiapas, Mexico, 2013

**DOI:** 10.1128/genomeA.01586-17

**Published:** 2018-01-25

**Authors:** Sandra Pérez-Agüeros, Joanna María Ortiz-Alcántara, Fabiola Garcés-Ayala, Edgar Mendieta-Condado, Elizabeth González-Durán, Nidia Aréchiga-Ceballos, Martin Melo-Munguía, Susana Chávez-López, Albert Sandoval-Borja, Mauricio Gómez-Sierra, Rita Terán-Toledo, David Martínez-Solís, Israel Animas-Vargas, Beatriz Escamilla-Ríos, Belem Torres-Longoria, Irma López-Martínez, Lucía Hernández-Rivas, José Alberto Díaz-Quiñonez, José Ernesto Ramírez-González

**Affiliations:** a Instituto de Diagnóstico y Referencia Epidemiológicos Dr. Manuel Martínez Báez, Mexico City, Mexico; b División de Estudios de Posgrado, Facultad de Medicina, UNAM, Mexico City, Mexico; c Escuela Nacional de Ciencias Biológicas, IPN, Mexico City, Mexico

## Abstract

Rabies virus (RABV), a member of the genus *Lyssavirus*, causes encephalitis that is almost always fatal following the onset of clinical signs. Here, we report the complete codifying sequence of an RABV isolated from a dog in Mexico. Molecular data showed that this strain belongs to the Chiapas lineage.

## GENOME ANNOUNCEMENT

Rabies virus (RABV) is a member of the genus *Lyssavirus* in the family *Rhabdoviridae*. Its genome is a single-stranded negative-sense RNA virus of approximately 12 kb that encodes five proteins, nucleoprotein (N), phosphoprotein (P), matrix protein (M), glycoprotein (G), and large protein (L) (1). Almost all human rabies deaths worldwide result from dog bites, most of them occurring in Africa and Asia, where millions of exposures occur annually and more than 50,000 people die each year as a result of the local unavailability of postexposure prophylaxis with vaccine and rabies immunoglobulin (2). In the past few decades in Mexico, massive and gratuitous canine vaccination campaigns (twice per year in March and September) combined with surgical sterilization of dogs and cats have reduced significantly the number of canine rabies cases, with no cases of human rabies transmitted by dogs reported since 2006. Despite a successful decrease in the number of cases of canine rabies, some enzootic circulation of rabies antigenic variant 1 (AgV1) still remains in localized areas in the south. Here, we present the complete genome sequence of RABV strain InDRE3944, isolated from a dog in Chiapas state, Mexico, in 2013.

RNA was extracted from dog brain tissue with a QIAmp viral RNA minikit (Qiagen), and four primer pairs were designed using the DRV-Mexico strain (GenBank accession number HQ450386) as a reference for the nucleotide positions. The SuperScript One-Step II reverse transcription-PCR (RT-PCR) Platinum Taq system (Invitrogen) was used to perform the reactions; the primers were RabF1a_2-28 (5′-CGCTTAACAACAAAATCASAGAAGAAG-3′), RabR1b_2980-3003 (5′-ACCAGATCCTGCCCTGAATATGAC-3′), RabF2a_2825-2851 (5′-GAACTGGGTATACAAGTTGAGRAGAAC-3′), RabR2b_5517-5540 (5′-AGGRGAGTTGAGATTGTAGTCAGA-3′), RabF3a_5334-5359 (5′-GTCATCTAAGCWTTTCAGTCGAGAAA-3′), RabR3b_8583-8609 (5′-GTTCTTGAGAGACTCCTYCTAAACTG-3′), RabF4a_8491-8515 (5′-CTCGATTTCTCAGTGAGCTCTTCAG-3′), and RabR4b_11769-11794 (5′-CAACTGTAGTCTAGIAGGGATGATCT-3′).

The RABV amplicons from InDRE3944 were sequenced with the Ion Torrent platform. A single-end library was generated, resulting in 171,280 reads (24,355,798 bases) with an average length of 142 bp. The resulting genome of 11,791 nucleotides was assembled with CLC Genomics Workbench version 7.5.1, using the DRV-Mexico strain as the mapping reference sequence (3). A single contig was obtained with an average coverage of 1,013×; it was annotated using Genome Annotation Transfer Utility (GATU) software (4) and submitted with the NCBI BankIt tool. In order to complete the full sequence, a pair of primers, RabEF_11744-11768 (5′-TCGAGGATATCAGATCTAGATCATC-3′) and RabER_11898-11924 (5′-ACGCTTAACAAAWAAACAATAAAGATG-3′), were designed to cover the entire genome, and the obtained amplicon was sequenced by the Sanger method. Sequence analysis identified 31 total amino acid (aa) differences compared with the DRV-Mexico strain (i.e., 1 aa difference in the N and M proteins, 5 aa differences in the P protein, 9 aa differences in the G protein, and 16 aa differences in the L protein). The strains differ in the length of the P proteins due to a double-stop codon in DRV-Mexico resulting in 296 aa; InDRE3944 encodes a protein of 297 aa. Preliminary phylogenomic analysis with virtual hybridization software was performed using the Universal Fingerprinting Chip 9-mer (UFC-9), allowing a single mismatch (5). This RABV isolate belongs to the Chiapas lineage, and previous analyses suggest recent dissemination events in dogs from Yucatan to Chiapas (6).

### Accession number(s).

The genome sequence of RABV strain InDRE3944 has been deposited in GenBank under the accession number KT006769. The version reported here is the second version, KT006769.2.
